# Pediatric suppurative parotitis caused by *Burkholderia pseudomallei*

**DOI:** 10.1186/s40409-016-0086-3

**Published:** 2016-11-09

**Authors:** Zengzhen Fu, Yingzi Lin, Qiang Wu, Qianfeng Xia

**Affiliations:** 1 Key Laboratory of Translational Medicine for Tropical Diseases (Hainan Medical University), Ministry of Education, Hainan Medical University, Haikou, 571199 China; 2 Department of Pediatrics, Hainan Maternity and Child Healthcare Hospital, Haikou, 571168 China; 3 Faculty of Tropical Medicine and Laboratory Medicine, Hainan Medical University, 3 Xue Yuan Road, Haikou, Hainan 570102 China

**Keywords:** Melioidosis, Parotitis, *Burkholderia pseudomallei*, Pediatrics

## Abstract

**Background:**

Suppurative parotitis caused by *Burkholderia pseudomallei* has been rarely found outside endemic areas.

**Case presentation:**

Herein, we report the recovery of *Burkholderia pseudomallei* from the pus of a suppurative parotitis observed in a 12-year-old boy who lived in Hainan province, China. Specimens of necrotic tissue were collected and sections were stained with hematoxylin and eosin. Pus sample was also collected for bacteriological examination. The suppurative inflammation was observed in the necrotic tissue section and *Burkholderia pseudomallei* were detected in the sample.

**Conclusion:**

In this adolescent, *Burkholderia pseudomallei* infection was present in the parotid, which consists of the first report of this bacterium in a parotitis case acquired in China.

## Background

Melioidosis, the disease caused by the bacterium *Burkholderia pseudomallei*, is endemic to Southeast Asia and northern Australia, and parts of South and Central America. Infection with *B. pseudomallei* can be contracted via several routes, including subcutaneous inoculation, ingestion, and likely inhalation [1]. Melioidosis is known as a ‘great mimicker’ since it may mimic several diseases from pyogenic bacterial infection to tuberculosis, and there is no pathognomonic sign of melioidosis [2–4]. The disease has an acute and a subacute forms and also a chronic relapsing state with associated high mortality. Risk factors for infection include diabetes, alcoholism, renal insufficiency and chronic steroid use [5]. Although melioidosis involves most organs, parotid involvement is rare. To the best of our knowledge, confirmed melioidosis parotitis was not previously reported except for a case that occurred after systemic melioidosis [6]. Therefore, we describe in this report the clinical presentation, management, and outcome of a child with primary melioidosis parotitis.

## Case presentation

The patient was a 12-year-old boy who lived in Dongfang county, Hainan province, a region located in the tropical area of China, who had never traveled abroad. He reported occasionally swimming in the lake of his hometown and had last did it roughly 1 month before the illness onset. This patient presented to the medical department with severe left parotid pain and a 4.0 × 3.5 cm swelling on the left face suggestive of parotitis. The swelling was soft and fluctuant and it was associated with tenderness, warmth and redness. There was no primary focus of infection found in the adjacent draining area. He had no related symptoms of fever, cough, expectoration or weight loss. The patient had no history of diabetes, renal disease, chronic lung disease or excessive alcohol consumption. He had normal vital signs. After parotid aspiration, he immediately received 2 g of ceftazidime intravenously twice a day together with 80 mg of gentamicin three times a day.

The investigation performed at our hospital revealed white blood cell count of 8.1 × 10^9^/L (69 % neutrophils), red blood cell count of 3.94 × 10^12^/L, platelet count of 231 × 10^9^/L, hematocrit of 34 %, hemoglobin concentration of 106 g/L, glucose concentration of 6.01 mmol/L, C-reactive protein concentration of 6.3 mg/L, and erythrocyte sedimentation rate was elevated (92 mmHg/1st hour).

**Fig. 1:**
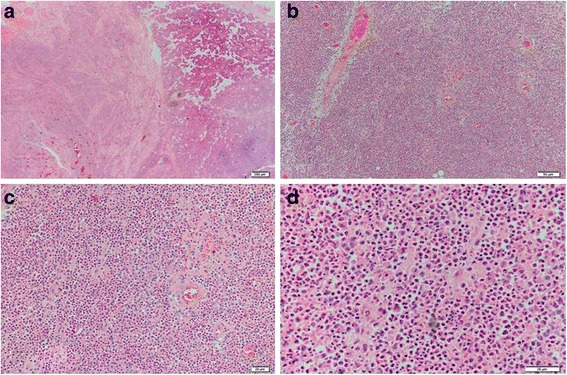
Histopathologic evaluation of parotid tissue specimens from the patient. Suppurative inflammation is observed in the parotid (**a**, **b**, **c** and **d**). Hematoxylin and eosin stain, original magnification: (**a**) 20×, (**b**) 100×, (**c**) 200× and (**d**) 400×. *Scale bars* indicate (**a**) 200 μm, (**b**) 50 μm, or (**c** and **d**) 20 μm

**Fig. 2:**
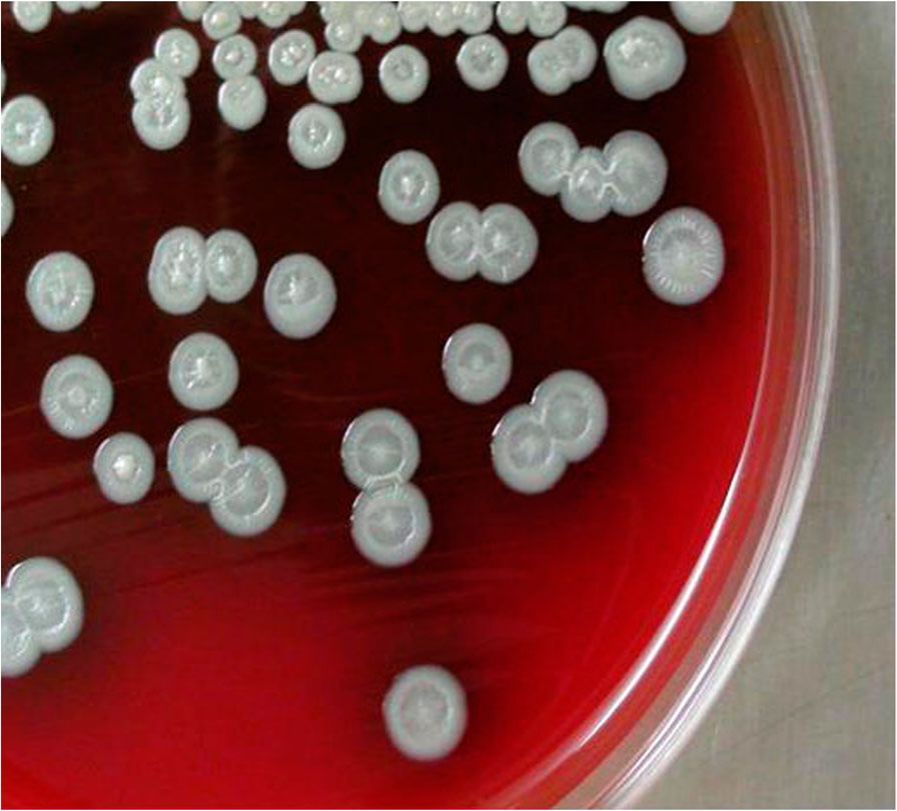
*Burkholderia pseudomallei* colonies on blood agar: they are creamy white with concentric ring-like pattern

For histologic analysis, specimens of necrotic tissue taken during the drainage operation were fixed in 4 % paraformaldehyde, sections were stained with hematoxylin and eosin, and suppurative inflammation was observed (Fig. 1). Pus was aspirated from the abscess for bacteriological examination. Subsequently, samples were inoculated on 5 % sheep blood agar and incubated at 37 °C in air. After 48 h, concentric rounded shaped and creamy white colonies were observed on the blood agar (Fig. 2). The isolate proved to be a non-fermenting gram-negative bacillus that was submitted to further identification using the VITEK-2 identification system (BioMérieux, France). VITEK-2 suggested that the isolate was *Burkholderia pseudomallei*, with an excellent level of identification (>99 %), which was confirmed by 16S rRNA gene sequencing. The 16S rRNA gene was amplified by PCR using the primers 27F (5-AGAGTTTGATCCTGGCTCAG-3) and 1525R (5-AAGGAGGTGATCCAGCC-3) [7]. Sequence similarity searches were performed using the BLAST algorithm implemented in the NCBI database.

Antibiotic susceptibility testing was performed by broth macrodilution methods. The isolate was susceptible to imipenem (MIC 2 μg/mL) and trimethoprim-sulfamethoxazole (TMP-SMZ) (MIC 2 μg/mL). It was resistant to the following antibiotics: ampicillin (MIC ≥ 32 μg/mL), amikacin (MIC ≥ 32 μg/mL), ceftriaxone (MIC ≥ 32 μg/mL), cefazolin (MIC ≥ 32 μg/mL), nitrofurantoin (MIC ≥ 32 μg/mL), gentamicin (MIC ≥ 32 μg/mL), cefepime (MIC ≥ 32 μg/mL), levofloxacin (MIC ≥ 32 μg/mL), ceftazidime (MIC ≥ 32 μg/mL), ciprofloxacin (MIC ≥ 32 μg/mL) and piperacillin/tazobactam (MIC ≥ 32 μg/mL). According to the laboratory data, the therapy was therefore changed to intravenous imipenem and oral TMP-SMZ for 4 weeks. The patient was discharged with an additional treatment of 12 weeks of oral TMP-SMZ.

## Conclusions

Although data are lacking with regard to the exact locations, eight human cases of melioidosis were registered around Hainan province [8]. In this study, we have described a case of *B. pseudomallei* suppurative parotitis in China. Our patient was from Dongfang district, which is situated in the tropical zone of China. This was essentially a locally acquired case, as this patient had never travelled to any other country. To our knowledge, this is the first description of *B. pseudomallei* in a parotitis patient acquired in China.

Best clinical judgment and focused microbiological investigations are very important for early diagnosis [9, 10]. In this case, cultures from pus grew *B. pseudomallei* and the blood cultures were negative. The clinicians should provide an early appropriate antibiotic treatment to reduce the risk of death caused by melioidosis. However, therapeutic options for the treatment of melioidosis are limited as *B. pseudomallei* is resistant to numerous antibiotics, including aminoglycoside, penicillin, narrow- and expanded-spectrum cephalosporin and macrolide. *B. pseudomallei* is generally susceptible to TMP-SMZ, broad-spectrum cephalosporin and carbapenem [11]. High-dose intravenous ceftazidime is currently the first antibiotic of choice for the recommended treatment of acute melioidosis [12]. In this case, the isolate was resistant to ceftazidime and most other antibiotics. It was only susceptible to imipenem and TMP-SMZ. Our patient recovered following treatment with intravenous imipenem and oral TMP-SMZ for 4 weeks, and 3 months of oral TMP-SMZ, as per the recommendations. He was doing well on follow-up. Intravenous imipenem and oral TMP-SMZ may prove efficacious for the treatment of suppurative parotitis caused by *B. pseudomallei*.

The presence of melioidosis may lead to several of deaths since most laboratories of Hainan have no experience in recognizing this organism. The cases are common enough that China is listed as one of the endemic countries for melioidosis. A high index of suspicion is needed to diagnose these complications, even in endemic regions like ours, as these complications can be life threatening, and melioidosis is not commonly identified as the source of severe presentations. Based on the present report, we believe that infections caused by *B. pseudomallei* should be included in the differential diagnosis of suppurative parotitis in Hainan, China.
