# Erratum to: Pediatric suppurative parotitis caused by *Burkholderia pseudomallei*

**DOI:** 10.1186/s40409-017-0095-x

**Published:** 2017-01-24

**Authors:** Zengzhen Fu, Yingzi Lin, Qiang Wu, Qianfeng Xia

**Affiliations:** 1 Key Laboratory of Translational Medicine for Tropical Diseases (Hainan Medical University), Ministry of Education, Hainan Medical University, Haikou, 571199 China; 2 Department of Pediatrics, Hainan Maternity and Child Healthcare Hospital, Haikou, 571168 China; 3 Faculty of Tropical Medicine and Laboratory Medicine, Hainan Medical University, 3 Xue Yuan Road, Haikou, Hainan 570102 China

## Erratum

Since the publication of our article [1], we have become aware [2] that cases of pediatric suppurative parotitis caused by *Burkholderia pseudomallei* have been previously reported. Therefore the statement “Although melioidosis involves most organs, parotid involvement is rare. To the best of our knowledge, confirmed melioidosis parotitis was not previously reported except for a case that occurred after systemic melioidosis [6]” is inaccurate. It should read :“Pediatric suppurative parotitis caused by *B. pseudomallei* has already been reported in Thailand by Dance et al. [3], who described 21 cases between 1986 and 1987, and in Cambodia by Stoesser et al. [4], who observed 39 cases of the disease between 2007 and 2011. To the best of our knowledge, this is the first report of locally acquired *B. pseudomallei* parotitis in Hainan, China.”

## References

[1] Fu Z, Lin Y, Wu Q, Xia Q (2016). Pediatric suppurative parotitis caused by Burkholderia pseudomallei. J Venom Anim Toxins Trop Dis.

[2] Dance D (2016). Meliodosis parotitis in children. J Venom Anim Toxins Trop Dis.

[3] Dance DA, Davis TM, Wattanagoon Y, Chaowagul W, Saiphan P, Looareesuwan S (1989). Acute suppurative parotitis caused by Pseudomonas pseudomallei in children. J Infect Dis.

[4] Stoesser N, Pocock J, Moore CE, Soeng S, Chhat HP, Sar P (2012). Pediatric suppurative parotitis in Cambodia between 2007 and 2011. Pediatr Infect Dis J.

